# Incremental Discriminant Analysis in Tensor Space

**DOI:** 10.1155/2015/587923

**Published:** 2015-08-03

**Authors:** Liu Chang, Zhao Weidong, Yan Tao, Pu Qiang, Du Xiaodan

**Affiliations:** ^1^College of Information Science and Technology, Chengdu University, Chengdu 610106, China; ^2^Key Laboratory of Pattern Recognition and Intelligent Information Processing in Sichuan, Chengdu 610106, China

## Abstract

To study incremental machine learning in tensor space, this paper proposes incremental tensor discriminant analysis. The algorithm employs tensor representation to carry on discriminant analysis and combine incremental learning to alleviate the computational cost. This paper proves that the algorithm can be unified into the graph framework theoretically and analyzes the time and space complexity in detail. The experiments on facial image detection have shown that the algorithm not only achieves sound performance compared with other algorithms, but also reduces the computational issues apparently.

## 1. Introduction

Nowadays, increasing amounts of data in the field of industrial, economic, medical, and other application areas, such as signals, measurements, images, and videos, are becoming available due to the development of computer technology. In order to excavate the hidden information in the data implicitly describing underlying processes or structures, advanced intelligent tools are proposed. However, since the stochastic nature of the processes and their measurement, structure in this data is mostly collected with noise. Consequently, it is reasonable to seek robust and adaptive tools that can cope with this nature.

Computational intelligence techniques have been investigated to answer this need. These techniques have been concerned with reproducing the abilities of human brains. Machine learning techniques exactly imitate the learning procedure of human, which construct learning model based on example data and use that to make predictions and decisions. However, due to the noise in data, it is important to construct efficient learning model to help sift useful information from the noise.

In regards to this, machine learning algorithms project high-dimensional data into low-dimensional feature space to make their low-features as separable as possible. Generally, they are classified into two categories: supervised learning and unsupervised learning. The essential difference between supervised learning and unsupervised learning is that whether the class information is considered. Generally speaking, the recognition performance of supervised learning is superior to that of unsupervised learning. As a classical machine learning algorithm, linear discriminate analysis (LDA) [1, 2] seeks optimal discriminative vectors to maximize the interclass scatter matrix and to minimize the intraclass scatter matrix. A large number of research works have shown the predominant advantage of LDA in various applications.

It is worth noting that traditional LDA is based on vector model. It requires all data being vectorized before learning. Actually, high-dimensional image data is structured data; the vectorization operation will break the correlation relationship of different pixels. Furthermore, the vectorization operation also is easy to result in the curse of dimensionality problem. As a result, machine learning algorithms [3–11] based on tensor algebra are investigated. These algorithms consider high-dimensional image as a high order tensor and introduce tensor algebra to analyze tensor data. Tensor representation not only is helpful to preserve the structure of high-dimensional image, but also serves as an effective way to avoid the curse of dimensionality problem. To unify all machine learning algorithms, [12] proposes the graph embedding framework. Under this graph embedding framework, two kinds of projective forms are summarized, called vector-to-vector and tensor-to-tensor forms, respectively.

However, for all machine learning algorithms, they have to train all samples again when new samples are added, which results in heavy computational cost. Consequently, incremental machine learning algorithms are proposed [13–17]. But most incremental learning algorithms focus on vector machine learning. Only a limited number of works study incremental learning in tensor space [18–20]. To investigate the incremental tensor learning, this paper develops incremental tensor discriminant analysis (ITDA), which employs supervised learning in tensor space and introduces incremental learning to process online learning. Furthermore, as a kind of machine learning algorithm, this paper also exploits the relationship between the proposed methods and the graph embedding framework and proves that the algorithm is a special case of tensor-to-tensor form under the graph embedding framework theoretically. This paper also analyzes the time and space complexity in detail. At last, this paper conducts facial image detection experiments to evaluate the proposed method. The experimental results have demonstrated the advantage of the method.

## 2. Tensor Discriminant Analysis

For multidimensional image data *X* = {*X*_1_,…, *X*_*K*_}, where *X*_*i*_ ∈ *ℝ* ^*I*_1_×⋯×*I*_*N*_^, the corresponding class label is *l*(*i*) ∈ [1, *C*], where *C* is the number of the class. Let the number of the *c*th class be *n*_*c*_; then the following definitions are introduced.

**Definition 1 .**

Within-class scatter tensor is defined:

(1) $$\begin{array}{c} S_{w}=\overset{C}{\underset{c=1}{\sum }}\;\overset{n_{c}}{\underset{i=1}{\sum }}{\left\|X_{i}-{\bar{X}}_{c}\right\|}^{2}, \end{array}$$

where ${\bar{X}}_{c}$ represent the mean tensor of the *c*th class.

**Definition 2 .**

Between-class scatter tensor is defined:

(2) $$\begin{array}{c} S_{b}=\overset{C}{\underset{c=1}{\sum }}n_{c}{\left\|{\bar{X}}_{c}-\bar{X}\right\|}^{2}, \end{array}$$

where $\bar{X}$ represent the total mean tensor.

**Definition 3 .**

Total scatter tensor is defined:

(3) $$\begin{array}{c} S_{t}=\overset{K}{\underset{i=1}{\sum }}{\left\|X_{i}-\bar{X}\right\|}^{2}. \end{array}$$

It is easy to derive that

(4) Sw+Sb=∑c=1C ∑i=1ncXi−X¯c2 +∑c=1CncX¯c−X¯2=∑c=1C ∑i=1ncvec⁡Xi−vec⁡X¯c2 +∑c=1Cncvec⁡X¯c−vec⁡X¯2=∑i=1Kvec⁡Xi−vec⁡X¯2=St.

**Definition 4 .**

Mode-*n* within-class scatter matrix is defined:

(5) $$\begin{array}{c} S_{w}^{\left(n\right)}=\overset{C}{\underset{c=1}{\sum }}\;\overset{n_{c}}{\underset{i=1}{\sum }}\left(X_{i}^{\left(n\right)}-{\bar{X}}_{c}^{\left(n\right)}\right){\left(X_{i}^{\left(n\right)}-{\bar{X}}_{c}^{\left(n\right)}\right)}^{T}, \end{array}$$

where *X*_*i*_^(*n*)^ is the mode-*n* matrix of the *i*th sample and ${\bar{X}}_{c}^{\left(n\right)}$ is the mode-*n* mean matrix of the *c*th class.

**Definition 5 .**

Mode-*n* between-class scatter matrix is defined:

(6) $$\begin{array}{c} S_{b}^{\left(n\right)}=\overset{C}{\underset{c=1}{\sum }}n_{c}\left({\bar{X}}_{c}^{\left(n\right)}-{\bar{X}}^{\left(n\right)}\right){\left({\bar{X}}_{c}^{\left(n\right)}-{\bar{X}}^{\left(n\right)}\right)}^{T}, \end{array}$$

where ${\bar{X}}^{\left(n\right)}$ is the mode-*n* total mean matrix.

**Definition 6 .**

Mode-*n* total scatter matrix is defined:

(7) St(n)=∑i=1KXin−X¯nXin−X¯nT=Sbn+Swn.

The basic idea of TDA is to seek *N* projective matrices to make within-class scatter tensor smaller and between-class scatter tensor larger. The objective function is

(8) JU(1),…,U(N) =arg max⁡∑c=1CncY¯c−Y¯2∑c=1C∑i=1ncYi−Y¯c2 =arg max⁡∑c=1CncX¯c−X¯×UnTnn=1N2∑c=1C∑i=1ncXi−X¯c×UnTnn=1N2.

In order to solve the above function, the iterative technique is adopted. It is assumed that the projective matrices {*U* ^(1)^,…, *U* ^(*n* − 1)^, *U* ^(*n* + 1)^,…, *U* ^(*N*)^} are known; then *U* ^(*n*)^ is solved as follows:

(9) JU(n) =arg max⁡∑c=1CncUnTX¯cn−X¯nU(−n)U−nT∑c=1C∑i=1ncUnTXin−X¯cnU−n·Xin−X¯cnTUn−1hhhhhhhhhhhh∑c=1CncUnTX¯cn−X¯nU(−n)·X¯cn−X¯nTUnhhhhhhhhhhhh·∑c=1C ∑i=1ncUnTXin−X¯cnU−nU−nThhhhhhhhhhhh∑c=1C∑i=1ncUnTXin−X¯cnU−n·Xin−X¯cnTUn−1,

where *U* ^(−*n*)^ = *U* ^(*N*)^ ⋯ ⊗*U* ^(*n*+1)^ ⊗ *U* ^(*n*−1)^ ⋯ ⊗*U* ^(1)^. Since *U* ^(−*n*)^ *U* ^(−*n*)^*T*^^ = *I*, so the above equation can be rewritten:

(10) JU(n)=arg max⁡∑c=1CncUnTX¯cn−X¯nX¯cn−X¯nTU(n)∑c=1C∑i=1ncUnTXin−X¯cnXin−X¯cnTU(n)=arg max⁡U(n)TSb(n)U(n)U(n)TSw(n)U(n).

Based on the basic concept of TDA and related matrix knowledge, we can get the following theorems.

**Theorem 7 .**

In tensor discriminant analysis, the mode-*n* intraclass scatter matrix is generally nonsingularity.

**Proof**

Defining the following matrix

(11) $$\begin{array}{c} H_{w}^{\left(n\right)}=\left[X_{1}^{\left(n\right)}-{\bar{X}}_{l(1)}^{\left(n\right)},\ldots ,X_{M}^{\left(n\right)}-{\bar{X}}_{l(M)}^{\left(n\right)}\right], \end{array}$$

where *M* is the number of samples, *l*(*m*) expresses the class label of the *m*th sample. Then the mode-*n* intraclass scatter matrix is represented:

(12) $$\begin{array}{c} S_{w}^{\left(n\right)}=H_{w}^{\left(n\right)}H_{w}^{{\left(n\right)}^{T}}, \end{array}$$

where *H*_*w*_^(*n*)^ ∈ *ℝ* ^*I*_*n*_×*D*^, *D* = ∏_*k*=1,*k*≠*n*_^*N*^ *I*_*k*_. Generally speaking, *I*_*n*_ ≪ *D*; then

(13) $$\begin{array}{c} \mathrm{rank}\left(H_{w}^{\left(n\right)}\right)=\min\left(I_{n},D\right)=I_{n}. \end{array}$$

Further, we can get

(14) $$\begin{array}{c} \mathrm{rank}\left(S_{w}^{\left(n\right)}\right)=\mathrm{rank}\left(H_{w}^{\left(n\right)}H_{w}^{{\left(n\right)}^{T}}\right)=\mathrm{rank}\left(H_{w}^{\left(n\right)}\right)=I_{n}. \end{array}$$

So the theorem is proved.

**Theorem 8 .**

Equation (8) can be unified into the graph embedding framework [12].

**Proof**

Based on the basic concept of tensor algebra, the numerator of (8) can be rewritten:

(15) ∑c=1C ∑i=1ncYi−Y¯c2 =∑c=1C ∑i=1ncvec⁡Yi−vec⁡Y¯c2.

Letting $y_{i}=\mathrm{vec}(Y_{i})\mathrm{vec}({\bar{Y}}_{c})=\left(1/n_{c}\right)\sum_{j=1}^{n_{c}}y_{j}$, then the above equation is written:

(16) ∑c=1C ∑i=1ncyi−1nc∑j=1ncyj2 =∑c=1C ∑i=1ncyi−1nc∑j=1ncyjyi−1nc∑j=1ncyjT =∑i=1KyiyiT−∑c=1C1nc∑li=lj=cyiyjT =∑i=1K ∑j=1KWij(w)yiyiT−∑i,j=1KWij(w)yiyjT =12∑i=1K ∑j=1KWij(w)yiyiT+12∑i=1K ∑j=1KWij(w)yiyiT  −12∑i,j=1KWij(w)yiyjT  −12∑i,j=1KWij(w)yiyjT =12∑i,j=1KWij(w)yiyiT+yjyjT−yiyjT−yjyiT =12∑i,j=1KWij(w)yi−yjyi−yjT =12∑i,j=1KWij(w)Yi−Yj2,

where

(17) $$\begin{array}{c} W_{ij}^{\left(w\right)}=\left\{\begin{array}{cc} \frac{1}{n_{c}}, & l\left(i\right)=l\left(j\right)=c,\\ 0, & l\left(i\right)\neq l\left(j\right). \end{array}\right. \end{array}$$

Within the low-dimensional feature space, it is desired to preserve the property as demonstrated in (4), so the denominator of (8) is formulated as follows:

(18) ∑c=1CncY¯c−Y¯2 =∑c=1Cncvec⁡Y¯c−vec⁡Y¯2 =∑i=1Kvec⁡Y¯i−vec⁡Y¯2  −∑c=1C ∑i=1ncvec⁡Yi−vec⁡Y¯c2 =∑i=1Kyi−y¯yi−y¯T  −∑c=1C ∑i=1ncvec⁡Yi−vec⁡Y¯c2,

where

(19) ∑i=1Kyi−y¯yi−y¯T=∑i=1KyiyiT−1K∑i,j=1KyiyjT=∑i=1K∑j=1K1KyiyiT−1K∑i,j=1KyiyjT=121K∑i,j=1KyiyiT+1K∑i,j=1KyiyiT−1K∑i,j=1KyiyjT−1K∑i,j=1KyiyjT=12∑i,j=1K1KyiyiT+yjyjT−yiyjT−yjyiT.

Combining (19) with (16), (18) can be written:

(20) ∑c=1CncY¯c−Y¯2 =12∑i,j=1K1K−Wij(w)yiyiT+yjyjT−yiyjT−yjyiT =12∑i,j=1KWij(b)yiyiT+yjyjT−yiyjT−yjyiT =12∑i,j=1KWij(b)yi−yjyi−yjT =12∑i,j=1KWij(b)Yi−Yj2,

where

(21) $$\begin{array}{c} W_{ij}^{\left(b\right)}=\frac{1}{n}-W_{ij}^{\left(w\right)}=\left\{\begin{array}{cc} \frac{1}{K}-\frac{1}{n_{c}}, & l\left(i\right)=l\left(j\right)=c,\\ \frac{1}{K}, & l\left(i\right)\neq l\left(j\right). \end{array}\right. \end{array}$$

Consequently, (8) is expressed:

(22) JU(1),…,U(N) =arg max⁡∑i,j=1KWij(b)Yi−Yj2∑i,j=1KWij(w)Yi−Yj2 =arg max⁡∑i,j=1KWijbXi−Xj×UnTnn=1N2∑i,j=1KWijwXi−Xj×UnTnn=1N2.

The form of (22) is consistent with the tensor-to-tensor form of the graph embedding framework. Therefore, (8) can be unified into the graph embedding framework.

## 3. Incremental Tensor Discriminant Analysis

### 3.1. Incremental Learning Based on a Single Sample

In order to distinguish these variables that need to be updated during incremental learning procedure, the paper employs the subscript old to mark the variables before incremental learning. For example, ${\bar{X}}_{\text{old}}$ expresses the total mean tensor before new samples are added.

When a single sample *X*_new_ is added, its class label is *l*_new_; then the mode-*n* total mean matrix becomes

(23) $$\begin{array}{c} {\bar{X}}^{\left(n\right)}=\frac{K{\bar{X}}_{\text{old}}^{\left(n\right)}+X_{\text{new}}^{\left(n\right)}}{K+1}. \end{array}$$

If *l*_new_ ∉ [1, *C*], that is, the new sample belongs to a new class. In this case, the total class number is *C* = *C*_old_ + 1 and mode-*n* interclass scatter matrix is updated:

(24) Sb(n)=∑c=1Cnc(X¯c(n)−X¯(n))X¯cn−X¯nT=∑c=1Coldnold_c(X¯old_c(n)−X¯(n))X¯old_cn−X¯nT +Xnewn−X¯nXnewn−X¯nT,

where *n*_*c*_ is the updated sample number of the *c*th class. Mode-*n* intraclass scatter matrix is

(25) Sw(n)=∑c=1C ∑i=1nc(Xi(n)−X¯c(n))Xin−X¯cnT=∑c=1Cold+1 ∑i=1nc(Xi(n)−X¯c(n))Xin−X¯cnT=∑c=1Cold ∑i=1nold_c(Xin−X¯old_cn)Xin−X¯old_cnT +Xnewn−X¯newnXin−X¯newnT,

where ${\bar{X}}_{\text{new}}^{\left(n\right)}$ is the mode-*n* mean matrix of the new sample. Because a single sample is added and it belongs to a new class, we can get

(26) $$\begin{array}{c} X_{\text{new}}^{\left(n\right)}={\bar{X}}_{\text{new}}^{\left(n\right)}. \end{array}$$

Then (25) becomes

(27) $$\begin{array}{c} S_{w}^{\left(n\right)}=\overset{C_{\text{old}}}{\underset{c=1}{\sum }}\;\overset{n_{\text{old}\_c}}{\underset{i=1}{\sum }}\left(X_{i}^{\left(n\right)}-{\bar{X}}_{\text{old}\_c}^{\left(n\right)}\right){\left(X_{i}^{\left(n\right)}-{\bar{X}}_{\text{old}\_c}^{\left(n\right)}\right)}^{T}=S_{\text{old}\_w}^{\left(n\right)}. \end{array}$$

It is demonstrated in (27) that mode-*n* intraclass scatter matrix will not change when a new sample with new class is added.

When the class label of the new sample *l*_new_ = *r* ∈ [1, *C*_old_], that is, the class label is not a new class. In this case, the total class number *C* = *C*_old_; then mode-*n* interclass scatter matrix is

(28) $$\begin{array}{c} S_{b}^{\left(n\right)}=\overset{C}{\underset{c=1}{\sum }}n_{c}\left({\bar{X}}_{c}^{\left(n\right)}-{\bar{X}}^{\left(n\right)}\right){\left({\bar{X}}_{c}^{\left(n\right)}-{\bar{X}}^{\left(n\right)}\right)}^{T}. \end{array}$$

Mode-*n* intraclass scatter matrix is

(29) Sw(n)=∑c=1C ∑i=1nc(Xi(n)−X¯c(n))Xin−X¯cnT=∑c=1Cold+1 ∑i=1nc(Xi(n)−X¯c(n))Xin−X¯cnT=∑c=1c≠rCold ∑i=1nold_c(Xin−X¯old_cn)Xin−X¯old_cnT +∑k=1nold_r+1(Xk(n)−X¯r(n))Xkn−X¯rnT.

Because the new sample belongs to the *r*th class, then the class mean of the *r*th class becomes

(30) $$\begin{array}{c} {\bar{X}}_{r}^{\left(n\right)}=\frac{n_{\text{old}\_r}{\bar{X}}_{\text{old}\_r}^{\left(n\right)}+X_{\text{new}}^{\left(n\right)}}{n_{\text{old}\_r}+1}. \end{array}$$

Based on this, we can get

(31) ∑k=1nold_r+1(Xk(n)−X¯r(n))Xkn−X¯rnT =∑k=1nold_r(Xk(n)−X¯r(n))Xin−X¯rnT  +(Xnew(n)−X¯r(n))Xnewn−X¯rnT =∑k=1nold_rXkn−X¯old_rn+X¯old_rn−X¯rn    ·Xkn−X¯old_rn+X¯old_rn−X¯rnT  +(Xnew(n)−X¯old_r(n)−Xnew(n)−X¯old_r(n)nold_r+1)  ·Xnewn−X¯old_rn−Xnewn−X¯old_rnnold_r+1T =∑k=1nold_r(Xk(n)−X¯old_r(n))Xkn−X¯old_rnT  +nold_rnold_r+1Xnewn−X¯old_rnXnewn−X¯old_rnT.

So (29) is simplified:

(32) Sw(n)=∑c=1c≠rCold ∑i=1nold_c(Xi(n)−X¯old_c(n))Xin−X¯old_cnT +∑k=1nold_r(Xk(n)−X¯old_r(n))Xkn−X¯old_rnT +nold_rnold_r+1(Xnewn−X¯old_rn)Xnewn−X¯old_rnT=∑c=1Cold ∑i=1nold_c(Xi(n)−X¯old_c(n))Xin−X¯old_cnT +nold_rnold_r+1Xnewn−X¯old_rnXnewn−X¯old_rnT.

### 3.2. Incremental Learning Based on Multisamples

When several samples are added, new added samples *X*_new_ = {*X*_*K*+1_,…, *X*_*K*+*T*_}, *T* ≥ 1, the corresponding class labels are *l*_new_ = {*l*_1_,…, *l*_*T*_}. Without loss of generality, it is assumed that *n*_new_*r*_ samples belong to the *r*th class; then the mean tensor of the *r*th class is updated:

(33) X¯r=1nold_r+nnew_r(nold_rX¯old_r+∑l(i)=ri=1nnew_rXi)=(nold_rX¯old_r+nnew_rX¯new_r)nold_r+nnew_r,

where ${\bar{X}}_{\text{new}\_r}$ is the mean tensor of the new samples belonging to the *r*th class. The corresponding mode-*n* mean matrix of the *r*th class is

(34) $$\begin{array}{c} {\bar{X}}_{r}^{\left(n\right)}=\frac{\left(n_{\text{old}\_r}{\bar{X}}_{\text{old}\_r}^{n}+n_{\text{new}\_r}{\bar{X}}_{\text{new}\_r}^{n}\right)}{n_{\text{old}\_r}+n_{\text{new}\_r}}. \end{array}$$

Then the number of samples in the *r*th is

(35) $$\begin{array}{c} n_{r}=n_{\text{old}\_r}+n_{\text{new}\_r}. \end{array}$$

The total mean tensor is updated:

(36) $$\begin{array}{c} \bar{X}=\frac{K{\bar{X}}_{\text{old}}+\sum_{i=K+1}^{K+T}X_{i}}{K+T}=\frac{K{\bar{X}}_{\text{old}}+T{\bar{X}}_{\text{new}}}{K+T}, \end{array}$$

where ${\bar{X}}_{\text{new}}$ is the mean tensor of all new samples. The interclass scatter mean tensor is updated:

(37) $$\begin{array}{c} S_{b}=\overset{C}{\underset{c=1}{\sum }}n_{c}{\left\|{\bar{X}}_{c}-\bar{X}\right\|}^{2}. \end{array}$$

The corresponding mode-*n* interclass scatter matrix is

(38) $$\begin{array}{c} S_{b}^{\left(n\right)}=\overset{C}{\underset{c=1}{\sum }}n_{c}\left({\bar{X}}_{c}^{\left(n\right)}-{\bar{X}}^{\left(n\right)}\right){\left({\bar{X}}_{c}^{\left(n\right)}-{\bar{X}}^{\left(n\right)}\right)}^{T}. \end{array}$$

The mode-*n* intraclass scatter matrix is

(39) Sw(n)=∑c=1C ∑i=1nc(Xi(n)−X¯c(n))Xin−X¯cnT=∑c=1C∑i=1nold_c(Xi(n)−X¯old_c(n))Xin−X¯old_cnThhhhhh∑i=1nold_c(Xi(n)−X¯old_c(n))Xin−X¯old_cnT+nold_c(X¯old_c(n)−X¯c(n))X¯old_cn−X¯cnT +∑c=1C∑i=1nnew_c(Xin−X¯new_cn)Xin−X¯new_cnThhhhhhhh∑i=1nold_c(Xi(n)−X¯old_c(n))Xin−X¯old_cnT+nnew_c(X¯new_cn−X¯cn)X¯new_cn−X¯cnT.

Substituting (34) into the following equation, we can get

(40) nold_c(X¯old_c(n)−X¯c(n))X¯old_cn−X¯cnT =nold_cX¯old_c(n)−nold_cX¯old_c(n)+nnew_cX¯new_c(n)nold_c+nnew_c  ·X¯old_c(n)−nold_cX¯old_c(n)+nnew_cX¯new_c(n)nold_c+nnew_cT =nold_cnnew_c2nold_c+nnew_c2(X¯old_c(n)−X¯new_c(n))X¯old_cn−X¯new_cnT =nold_cnnew_c2nc2(X¯old_c(n)−X¯new_c(n))X¯old_cn−X¯new_cnT.

Similarly, we can get

(41) nnew_c(X¯new_c(n)−X¯c(n))X¯new_cn−X¯cnT =nnew_cnold_c2nold_c+nnew_c2(X¯old_cn−X¯new_c)  ·X¯old_cn−X¯new_cT =nnew_cnold_c2nc2(X¯old_cn−X¯new_c)  ·X¯old_cn−X¯new_cT.

Substituting (40) and (41) into (39), we can obtain

(42) Sw(n)=∑c=1n∑i=1nold_c(Xi(n)−X¯old_c(n))Xin−X¯old_cnThhhhhhh∑i=1nold_c(Xi(n)−X¯old_c(n))Xin−X¯old_cnT+nold_c(X¯old_c(n)−X¯c(n))X¯old_cn−X¯cnT +∑c=1n∑i=1nnew_c(Xi(n)−X¯new_c(n))Xin−X¯new_cnThhhhhhh∑i=1nnew_cXin−X¯newcnXin−X¯newcnT+nnew_cX¯new_cn−X¯cnX¯new_cn−X¯cnT=∑c=1n∑i=1nold_c(Xin−X¯old_cn)Xin−X¯old_cnThhhhhh∑i=1nold_c(Xi(n)−X¯old_c(n))Xin−X¯old_cnT+∑i=1nnew_cXin−X¯new_cnXin−X¯new_cnT +∑c=1nnold_cnnew_cnc(X¯old_c(n)−X¯new_c(n))hhhhhhhh·X¯old_cn−X¯new_cnT.

Without loss of generality, it is supposed that, for *T* new samples, there are *n*_*C*+1_ samples belonging to the new class label *C* + 1; then updated mode-*n* interclass scatter matrix is

(43) Sb(n)=∑c=1ColdncX¯cn−X¯nX¯cn−X¯nT +nC+1X¯C+1n−X¯nX¯C+1n−X¯nT=∑c=1C+1ncX¯cn−X¯nX¯cn−X¯nT

and mode-*n* intraclass scatter matrix is

(44) Sw(n)=∑c=1Cold ∑i=1nc(Xi(n)−X¯c(n))Xin−X¯cnT +∑i=1nC+1(Xi(n)−X¯C+1(n))Xin−X¯C+1nT.

It is not difficult to find that incremental learning based on singular sample only is a special case of incremental learning based on multisample.

### 3.3. The Complexity Analysis

For tensor discriminant analysis, the main computational time is spent on the computation of interclass mean, total mean, inter- and intraclass scatter tensor, and Eigen decomposition. The computation cost of inter- and intra-class scatter tensors depends on the number of training samples. If there are a large number of training samples, it cannot avoid to increment computational time.

For incremental discriminant analysis, the main computational time is spent on the computation of updated inter- and intraclass scatter matrix and the class number.

For Eigen decomposition, both the time complexity of TDA and ITDA are *O*(*NI* ^3^). The main difference of the time complexity is the computation of inter- and intraclass scatter matrix. For TDA, the time complexity is *O*(*MNI* ^*N*+1^), so the time complexity will increase with the number of training samples. For ITDA, the time complexity is *O*(*TNI* ^*N*+1^ + *CNI* ^*N*+1^), which is related to the class number and the number of new samples. It has no relationship with the number of initial training samples. Consequently, ITDA is helpful to reduce the time complexity.

Considering the space complexity, ITDA is also superior to TDA. When new samples are added, TDA needs *M*∏_*n*=1_^*N*^ *I*_*n*_ bytes to save all training samples, but ITDA only needs ∏_*n*=1_^*N*^ *I*_*n*_ bytes to save new added samples, ∏_*n*=1_^*N*^ *I*_*n*_ bytes to save the total mean, *C*∏_*n*=1_^*N*^ *I*_*n*_ bytes to save the class mean, and *N*∑_*n*=1_^*N*^ *I*_*n*_^2^ bytes to save mode-*n* scatter matrix. Hence ITDA has the capability to save space.

Compared to incremental learning based on single sample with incremental learning based on multisamples, incremental learning based on single samples has an advantage to reduce the space complexity because it only deals with one sample for each time.

## 4. Experiments

**Figure 1:**
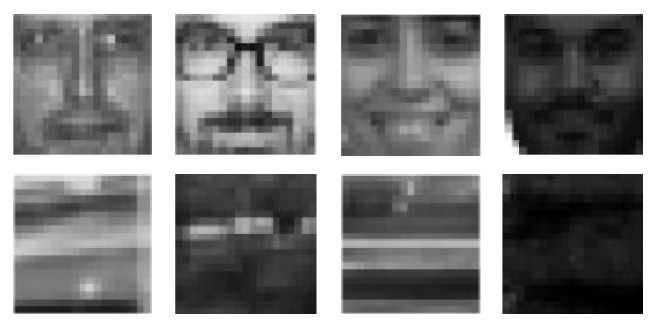
The samples of CBCL dataset.

In this section, a series of experiments are carried out to validate the performance of incremental tensor discriminant analysis (ITDA). The CBCL image data set is used to conduct facial image detection experiments. The dataset contains two classes of images, including facial images and nonfacial images as shown in Figure 1. The total number of the datasets is 2988 images, in which there are 2429 facial images and 559 nonfacial images. For each image, the size is 19 × 19. This paper divides whole dataset into training dataset with 1215 facial images and 280 nonfacial images and testing dataset with 1214 facial images and 279 nonfacial images. Furthermore, training dataset is divided into initial training dataset with 1015 facial images and 80 nonfacial images and four incremental datasets. Each incremental dataset has 50 facial images and 50 nonfacial images.

ITLDA integrates the tensor representation and incremental learning; it is reasonable to believe that it has the advantage to improve the detection performance and reduce the time and space complexity. In this respect, ITLDA is compared with LDA [21], ILDA [14], TPCA [22], ITPCA [23], and TDA [9]. LDA is the classical linear discriminant analysis. ILDA is the incremental version of LDA. TPCA is also called MPCA (multilinear principal component analysis), which carries on principal component analysis with tensor data. ITPCA is proposed to suit for incremental principal component analysis for tensor data. TDA also represents data as tensor structure and conducts multilinear discriminant analysis. For each time of incremental learning, the paper adds one incremental dataset and then extracts low-dimensional features on testing dataset. The nearest neighbor classifier is employed to classify these low-dimensional features.

**Figure 2:**
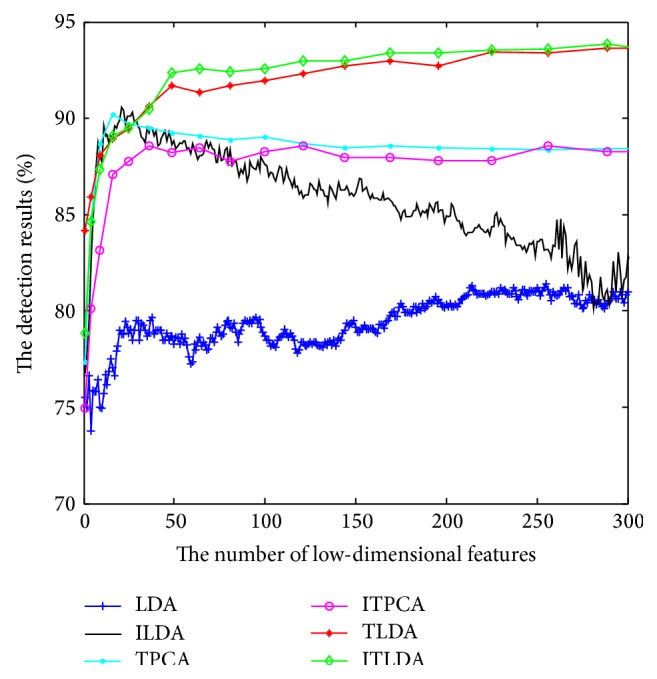
The detection results after the first incremental learning.

**Figure 3:**
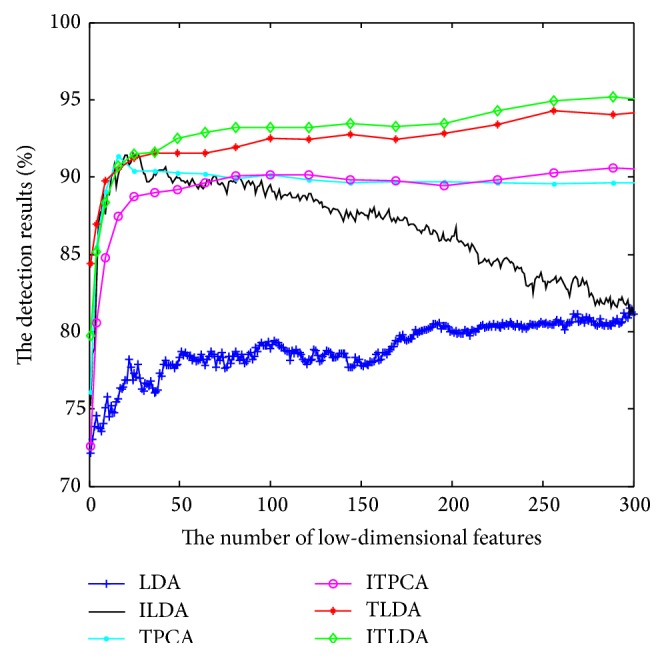
The detection results after the second incremental learning.

**Figure 4:**
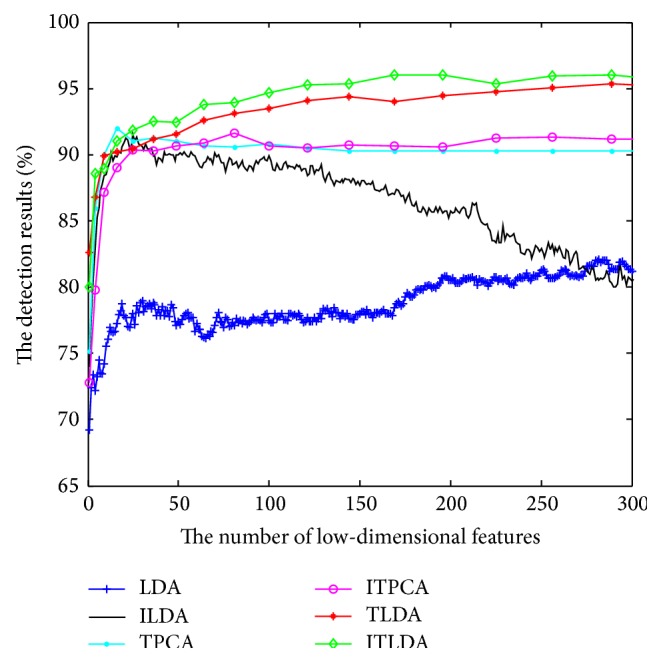
The detection results after the third incremental learning.

**Figure 5:**
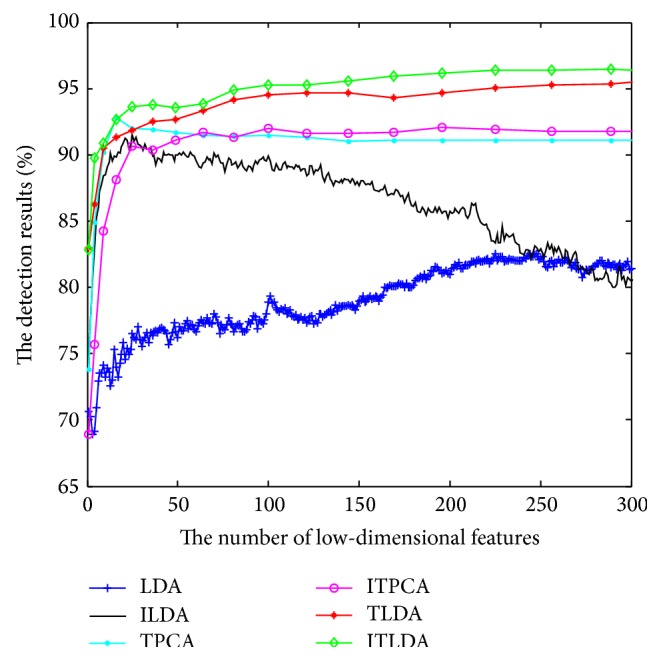
The detection results after the fourth incremental learning.

**Figure 6:**
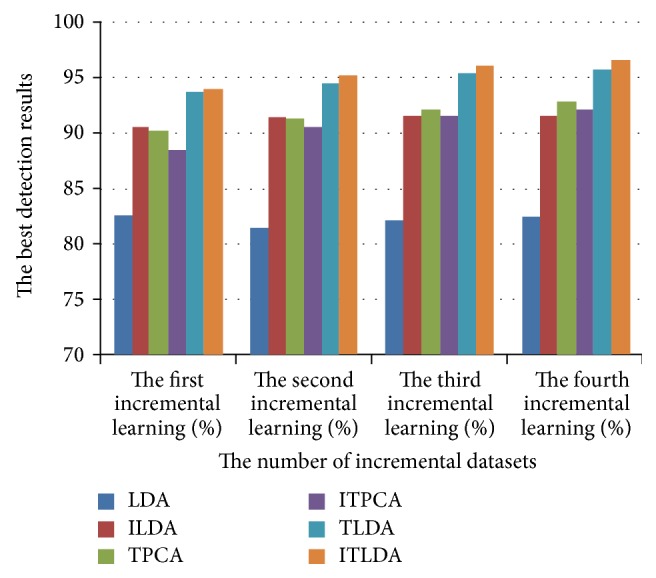
The comparison of the best detection results for different algorithms with incremental learning.

**Figure 7:**
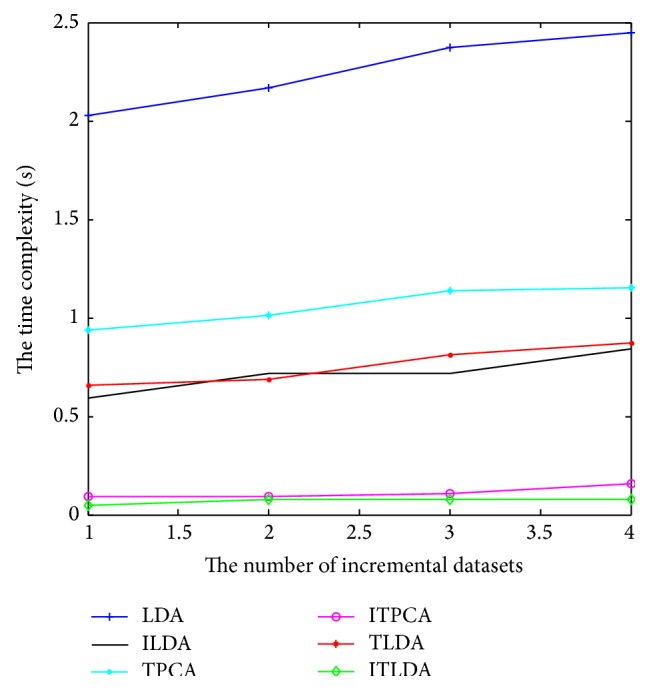
The comparison of time complexity.

**Figure 8:**
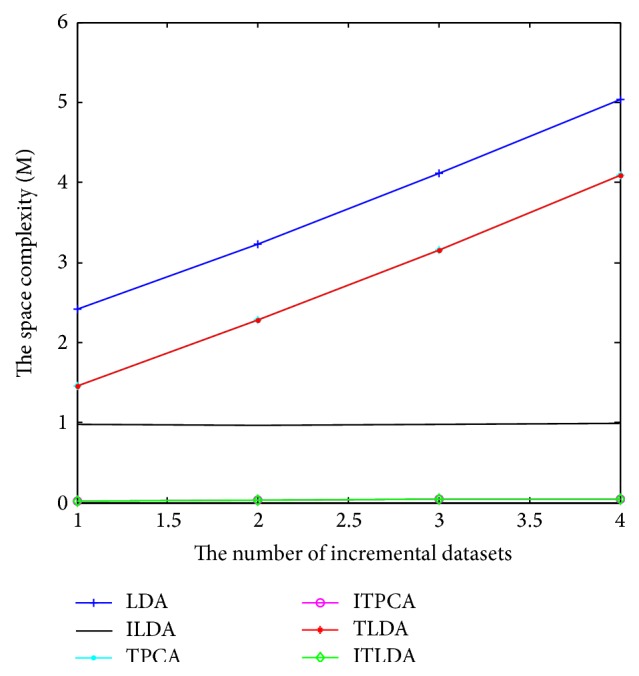
The comparison of space complexity.

The comparisons of detection performance for different algorithms with incremental learning are shown in Figures 2, 3, 4, and 5, respectively. It is worth noting that LDA is the worst and ILDA is better than LDA. However the detection results of ILDA drop with the increment of the dimension of low-dimensional features. TPCA and ITPCA have similar detection results and both of them exceed LDA and ILDA. The probable reason is that TPCA and ITPCA represent data as tensor structure, which make full use of the interior structure information to enhance the detection performance. TDA is superior to the above four algorithms. When the dimension of low-dimensional features is low, TDA and ITDA have comparative detection percent and ITDA begins to surmount TDA when the dimension of low-dimensional features increases.  Figure 6 and Table 1 have shown the best detection results of different algorithms. It can be seen that the detection performances of different algorithms are improved with the increment of incremental learning numbers and ITLDA always has the best performance. Consequently, it can be derived that the increment of incremental learning number is helpful to improve the detection result. More than that, as shown in Figures 7 and 8, incremental learning algorithms ILDA, ITPCA, and ITDA have the capability to alleviate time and space complexity apparently compared with nonincremental learning. Furthermore, since ITPCA and ITDA adopt tensor representation, they have lower time and space requirements than LDA.

## 5. Conclusions

In this paper, incremental tensor discriminant analysis (ITDA) is investigated. It adopts tensor representation to keep the structure information for high-dimensional images and introduces incremental learning to complete online learning. This paper also proves the relationship between ITDA and the graph framework theoretically. The facial detection experiments have shown that ITDA has better performance than TDA and is able to reduce the time and space complexity apparently.

## Figures and Tables

**Table 1:** The best detection results of different algorithms with incremental learning.

Algorithms	The first incremental learning (%)	The second incremental learning (%)	The third incremental learning (%)	The fourth incremental learning (%)
LDA	82.59	81.51	82.12	82.52
ILDA	90.56	91.49	91.56	91.56
TPCA	90.22	91.36	92.03	92.83
ITPCA	88.55	90.56	91.63	92.1
TLDA	93.67	94.44	95.38	95.78
ITLDA	93.83	95.17	96.05	96.45
